# The “Dark Side” of Pneumoperitoneum and Laparoscopy

**DOI:** 10.1155/2021/5564745

**Published:** 2021-05-19

**Authors:** Giuseppina Rosaria Umano, Giulia Delehaye, Carmine Noviello, Alfonso Papparella

**Affiliations:** Department of Woman, Child, and General and Specialized Surgery, University of Campania Luigi Vanvitelli, Largo Madonna Delle Grazie N. 1, Naples 8013, Italy

## Abstract

Laparoscopic surgery has been one of the most common procedures for abdominal surgery at pediatric age during the last few decades as it has several advantages compared to laparotomy, such as shorter hospital stays, less pain, and better cosmetic results. However, it is associated with both local and systemic modifications. Recent evidence demonstrated that carbon dioxide pneumoperitoneum might be modulated in terms of pressure, duration, temperature, and humidity to mitigate and modulate these changes. The aim of this study is to review the current knowledge about animal and human models investigating pneumoperitoneum-related biological and histological impairment. In particular, pneumoperitoneum is associated with local and systemic inflammation, acidosis, oxidative stress, mesothelium lining abnormalities, and adhesion development. Animal studies reported that an increase in pressure and time and a decrease in humidity and temperature might enhance the rate of comorbidities. However, to date, few studies were conducted on humans; therefore, this research field should be further investigated to confirm in experimental models and humans how to improve laparoscopic procedures in the spirit of minimally invasive surgeries.

## 1. Introduction

Laparoscopy has progressively substituted abdominal open surgery for many procedures as it has several advantages compared to laparotomy, even in children. In particular, laparoscopy is associated with better cosmetic results, shorter hospital stays, lower postoperative pain, and a faster return to daily activities [1]. However, it is not free from short- and long-term comorbidities, such as light pain, abdominal discomfort, and adhesion development [2–4].

The pneumoperitoneum is essential for laparoscopic surgery, as it enables visibility and mobility in the working site. Nevertheless, the pneumoperitoneum modifies abdominal-cavity homeostasis and might promote metabolic changes through mechanical and biochemical effects [5–8].

Historically, several gases were used to induce a pneumoperitoneum with the aim of reducing the incidence of local and systemic adverse effects. Helium and argon are inert gases that are compromised by low solubility. Evidence reported an increased rate of subcutaneous emphysema and venous gas embolism after helium insufflation [9]. Conversely, carbon dioxide (CO_2_) has 50-fold higher solubility compared to helium. Argon, like helium, is almost insoluble in blood, so it is associated with a higher risk of embolism. In addition, it induces cardiac depression [10]. Nitrous oxide was the preferred gas during the 1970s, but it is not used in current practice because of the risk of explosion. In fact, nitrous oxide is highly flammable and causes explosion during electrocautery [5].

On the basis of this evidence, CO_2_ has become a more suitable gas for pneumoperitoneum creation, as it is cost-effective, noncombustible, and soluble, and it is rapidly excreted with respiration. However, recent studies suggest that the use of CO_2_ during laparoscopic surgery is not free from adverse effects. The use of this gas might induce metabolic, immune, and structural modifications that can have clinical impact [11, 12].

Previous papers have shown that chemical, physical, and biological features of pneumoperitoneum might affect clinical outcomes, such as postoperative pain, length of stay, and recovery time [13–18].

These data suggest that chemical, physical, and biological effects of CO2 on peritoneum cause inflammation and tissue modifications [19]. In particular, we have seen that the grade of morphological changes is related to the level of intrabdominal pressure. CO2 pneumoperitoneum evokes an inflammatory response by modifying factors such as plasmatic and peritoneal chemokines levels [20].

To further investigate these aspects of laparoscopy, we reviewed the current research with regards to animal and human studies investigating peritoneal and systemic alterations during and after carbon dioxide pneumoperitoneum.

## 2. Materials and Methods

**Figure 1:**
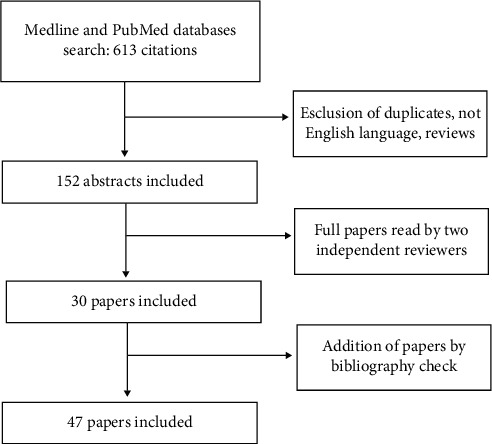
Flowchart of papers selection.

We performed a narrative search of Medline and PubMed databases with terms covering “laparoscopy”, “pneumoperitoneum”, “inflammation”, “mesothelium”, “ischemia–reperfusion”, and “adherence” published between January 1993 and April 2021. These terms were used in different combinations. Only English language papers were included. Articles were evaluated for scientific relevance and pertinence to the effect of pneumoperitoneum on peritoneal homeostasis impairment. Relevant references from selected papers were included. Study selection was executed by two authors; if a disagreement occurred, it was solved through analysis with the other coauthors (Figure 1).

## 3. Results

**Figure 2:**
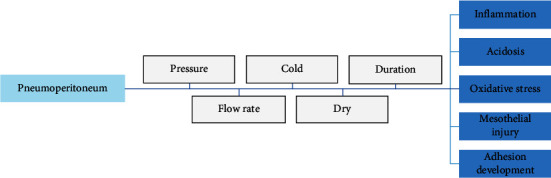
Pneumoperitoneum characteristics and related peritoneal effects.

The literature search revealed 621 articles. The majority of the selected papers investigated pneumoperitoneum effects in animal models: rats (*n* = 21), pigs (*n* = 4), rabbits (*n* = 1), and dogs (*n* = 1). Few papers were based on human studies (*n* = 20). Here, we report the results according to biological, metabolic, and histological alterations.  Figure 2 summarizes the effects of pneumoperitoneum and its variables on the peritoneal environment.

### 3.1. Inflammation

Intentional or accidental peritoneal injury induces a local acute phase reaction, namely, inflammation [21]. Inflammatory response is characterized by cellular infiltration and the release of proinflammatory cytokines such as IL-6, IL-1, and TNF-*α*. Surgery acts as local trauma and leads to both local and systemic inflammation. Laparoscopy was shown to produce a less severe peritoneal reaction compared to that of an open approach [22, 23]. Therefore, mini-invasive surgery is associated with lower pain and postoperative hospital stay [24–26]. However, pneumoperitoneum parameters might be modulated during laparoscopy with the aim of reducing early changes related to CO_2_ insufflation. In 2007, Papparella et al. reported that peritoneal insufflation with CO_2_ in rats was associated with eosinophils, mastocytes, and macrophages 2 h after laparoscopic surgery. The cellular response was also detectable at 24 h afterwards. The authors reported that peritoneal modification was lower in CO_2_ compared to the room-air insufflation group; nevertheless, carbon dioxide pneumoperitoneum resulted in an inflammatory response with respect to the control group [19]. Similar results were described by Ure et al. in pigs undergoing laparoscopy with room-air insufflation or CO_2_ pneumoperitoneum. Carbon dioxide pneumoperitoneum induced a lower release of IL-6 compared to that of air, whereas no significant difference for IL-1 and TNF-*α* levels was found [27].

In addition to gas type, insufflation pressure was reported to affect peritoneal mucosa integrity and cellular infiltration. In a rat model, low pneumoperitoneum pressure was associated with less intense cellular inflammatory response in terms of eosinophil granulocyte, macrophage, mast-cell, and lymphocyte composition compared to high pneumoperitoneum pressure 24 h after surgery. Therefore, there is a clear relationship between the grade of mucosa inflammation and level of pressure [28]. Recently, similar findings were observed by Poerwosusanta et al. in rats exposed to 8, 10, and 12 mmHg pneumoperitoneum. The authors found a progressive and significant increase in mast-cell peritoneal infiltration and degranulation with an increase in pressure 7 days after surgery [29]. Paralleling with local acute response, gas pressure was described as a factor influencing systemic inflammation. In fact, lower pneumoperitoneum pressure has been associated with less intense inflammatory cellular response after laparoscopy in subjects undergoing colorectal surgery [30]. Moreover, higher plasma TNF-*α* levels were found in 10 mm Hg pneumoperitoneum rats compared to those in 6 mm Hg and control groups. The three groups showed different IL-6 plasma levels, but the difference was not statistically significant [20]. Furthermore, the same authors showed systemic and local modifications of Rantes/CCL5 and MCP1/CCL2 that could have a role in these changes. The authors postulated that modifying these factors could make laparoscopic surgery less invasive. To date, there are few available studies investigating the relation between pneumoperitoneum features and inflammatory response in human models. Some evidence highlights lower cytokine release, stress-hormone levels, and plasma inflammatory markers after laparoscopy compared to laparotomy in both adults and children [31, 32]. Therefore, we need more studies to confirm the experimental findings about pneumoperitoneum influence in human inflammatory response.

### 3.2. Acidosis

Carbon dioxide induces the acidification of fluids, as it has the potential to induce carbonic acid reaction that dissociates in bicarbonate (HCO3-) and hydrogen ions (H+) [33]. This phenomenon is well-recognized to occur in blood and cerebrospinal fluid [33]; therefore, several experimental studies investigated the potential ability of CO_2_ insufflation in inducing peritoneal acidosis. An experimental study in dogs reported that CO_2_ abdominal insufflation induced acute peritoneal pH reduction after 10–20 minutes. This acidosis was also detectable in the blood, whereas laparotomy was not associated with any pH modification during and after surgery [34].

Wong et al. compared the insufflation effect of different gas types on pH modifications in pigs. They found that only CO_2_ was associated with acute peritoneal acidosis, whereas nitrous oxide and helium were not. In addition, peritoneal acidification was more severe with higher-pressure insufflation (12–15 versus 5–8 mmHg) [35]. Similarly, Hanly et al. found no abdominal acidification after helium or air insufflation and concluded that peritoneal pH changes during laparoscopy are induced by the specific activity of CO_2_ and not by mechanical abdominal distention [36]. The insufflation effect of different gases in rats was also investigated by Kuntz and colleagues [37]. They evaluated pH modifications in the blood, subcutaneous fat tissue, and the abdominal cavity after insufflation of CO_2_, air, and helium at different intra-abdominal pressure levels (0, 3, 6, and 9 mmHg). The authors reported that the CO_2_ effect differed according to site. In particular, CO_2_ significantly decreased blood and subcutaneous pH compared to air and helium. The influence of pressure on pH reduction was more effective than that of pneumoperitoneum length [37].

Conversely, an experimental study in rats exposed to different pressure levels and insufflation rates failed to find significant effects on peritoneal ph. In fact, pH rapidly decreased in the site of manipulation independently of pressure and insufflation rates. Moreover, acidification was only identified in areas directly exposed to the gas, whereas distal areas showed slight and not significant pH reduction [38].

Other physical CO_2_ characteristics were shown to induce pH changes in the abdominal cavity. An experimental swine-model compared peritoneal pH in four different groups: standard laparoscopy, laparoscopy with heated and humidified gas, laparoscopy with the administration of heated humidified gas and bicarbonate, and laparotomy. All laparoscopy groups displayed peritoneal acidification, whereas laparotomy did not. The addition of bicarbonate was ineffective in contrasting CO_2_ acidification. Conversely, the heated and humidified group showed lower pH reduction compared to that of standard laparoscopy. Heating and humidification could enhance local vascularization and subsequently CO_2_ peritoneal uptake [39].

Nevertheless, the investigation of peritoneal acidosis during laparoscopy is an interesting field of research, as it might be associated with local changes of immune response that could benefit the patient. In fact, evidence reported changes of cytokine levels, namely, IL-10 increase and TNF-*α* decrease, in response to bacterial lipopolysaccharides after CO_2_ insufflation [40]. Moreover, acidosis reduces macrophage activation [41]. Therefore, by modulating CO_2_ insufflation, it is possible to influence the inflammatory response and peritoneal homeostasis.

### 3.3. Oxidative Stress

During laparoscopy, CO_2_ insufflation in the abdominal cavity is necessary to visualize the working space; pressure and gas insufflation rate have a pivotal role in this phenomenon. However, increased intra-abdominal pressure might compromise splanchnic perfusion and capillary microcirculation and lead to reduced oxygen content and related ischemia–reperfusion injury with the consequent production of reactive oxygen species (ROS) [42–47].

ROS release, microcirculation impairment, and tissue hypoxia are the first steps of peritoneal injury, ranging from mesothelial denudation to inflammation and adhesion development [48]. Therefore, efforts in reducing oxidative-stress damage might be encouraged to prevent long-term laparoscopy-related comorbidities. Several animal and human studies indicated the role of pneumoperitoneal length and pressure in ischemia–reperfusion damage. Molinas et al. reported that tissue hypoxia increased with longer pneumoperitoneal length and higher insufflation rate in a murine model. These parameters were associated with adhesion development, and the addition of 2%–3% oxygen to insufflation gas was effective in reducing peritoneal fibrosis [49]. Accordingly, low intra-abdominal pressure did not affect peritoneal-tissue oxygen tension during laparoscopy in rats compared to laparotomy, confirming that less pressure causes minor damage to capillary flow and consequently tissue injury [50]. In particular, other authors reported that pressure of at least 12 mmHg significantly reduces splanchnic blood flow in rats [51].

Contrasting results were reported by Giannotti and coworkers, who failed to find compromised gut partial tissue oxygen tension during laparoscopy for left colon resection versus laparotomy [52]. Laparoscopy was conversely associated with an increased tissue oxygen tension that was equally decreased after mesenterial traction in laparoscopy and laparotomy groups. However, the authors observed a rise in plasmatic markers of oxidative stress with 15 mmHg pneumoperitoneal pressure [52]. Previous studies reported similar findings about the increase in oxidative plasma markers after laparoscopy. In 1998, Taskin et al. reported changes in free radical scavengers, namely, glutathione, glutathione peroxidase, superoxide dismutase, and catalase in 28 women undergoing operative laparoscopy. Modifications were directly associated with CO_2_ exposure length and insufflation rate [53]. Similarly, Polat reported that 15 mmHg pressure was associated with a significant increase in plasmatic malondialdehyde, a maker of lipid peroxidation, compared to 10 mmHg pneumoperitoneum [54]. Moreover, recently, Poerwosusanta et al. showed that H_2_O_2_ and malondialdehyde serum levels, as a direct expression of oxidative stress, significantly increased with the rise of insufflation pressure in rats. In addition, the levels of catalase and superoxide dismutase decreased in terms of oxidative-stress index. These changes were correlated with cellular inflammation and mesothelial-cell abnormalities [29].

In addition to minimizing intra-abdominal pressure, ischemic preconditioning was investigated as a method to reduce laparoscopy-induced oxidative stress. It consists of short ischemia–reperfusion cycles during early laparoscopy that might mitigate reoxygenation injury. Several experimental animal models ruled out the ability of preconditioning in reducing oxidative-stress injury in organs and plasma [55–58].

To date, there have been only two studies investigating ischemic preconditioning in humans. Kolbenschlag J et al. performed remote ischemic conditioning consisting of cycles of ischemia–reperfusion applied to the upper arm that were followed by improvement in skin microcirculation in the leg of 60 healthy volunteers [59]. More interesting are the results from the study of Veres and colleagues. They randomized 30 patients undergoing laparoscopic surgery to standard therapy and preconditioning and evaluated changes in plasmatic oxidative-stress markers. The ischemic preconditioning group showed significant improvement in glutathione levels and a significant reduction in pain according to the visual analog scale (VAS). Therefore, the authors concluded that this modification of pneumoperitoneum induction is a promising tool in limiting oxidative stress during laparoscopy [60]. However, more studies are needed to confirm this extremely preliminary evidence.

### 3.4. Mesothelial Modifications

The peritoneum is constituted by a single layer of mesothelial cells with an underlying basal membrane and connective tissue containing immune cells, fibroblasts, collagen, and blood vessels [61, 62]. It is involved in the regulation of several biologic processes, such as inflammation, angiogenesis, fibrinolysis, and tissue remodeling. Any kind of mechanical and chemical insult activates inflammation and fibrotic tissue deposition [62]. Therefore, the preservation of the mesothelial lining is crucial for reducing postsurgical adhesions. Animal studies described mesothelium changes during and after a pneumoperitoneum with electron microscopy. Volz and coworkers found cellular retraction and bulging up, intercellular clefts, and the exposition of basal lamina from 60 to 120 minutes after pneumoperitoneum release. These alterations involved the entire abdominal cavity and worsened until 12 hours after surgery. Mesothelial integrity was restored 2 days after surgery [63]. Similar findings were proposed by Suematsu et al., who compared mesothelial characteristics after the insufflation of different gases, namely, helium, air, and carbon dioxide. CO_2_ induced early cellular bulging up that reverted after 24 h. However, intercellular clefts and inflammatory infiltration were detectable at 24 h. Helium pneumoperitoneum was associated with more severe and persistent alterations [64]. Conversely, in another murine experimental model, peritoneal changes were not associated with gas type, whereas morphological peritoneal modifications were dependent on increased pneumoperitoneum period, insufflation flow, and pressure [65]. In addition, gas characteristics in terms of temperature and humidity were related to peritoneal injury. Standard pneumoperitoneum is induced with cold and dry gas [5, 6, 66] that induces dehydration and morphological alterations of the peritoneum. Therefore, the use of heated humidified gas was proven to prevent intercellular clefts [9], basal lamina exposure [67], and adhesion formation [68]. In contrast, in the study of Hazebroek et al., the effect on the mesothelial layer was independent from CO_2_ temperature and humidity [69]. However, previous findings were confirmed by Sampurno et al. who performed randomized laparoscopic rectal resection in 16 pigs to receive dry–cold or humidified–heated CO_2_. The dry–cold group showed significant early mesothelial modifications, such as cellular retraction and bulging, and microvillus alterations compared to the humidified–heated group [70]. Similarly, another experimental swine-model study reported reduced mesothelial thickness in pigs undergoing high-pressure pneumoperitoneum 7 days after laparoscopy. Damage was directly correlated with the increase in pressure and mast-cell infiltration. Mast-cell activation and degranulation could lead to the release of metalloproteinases that mediate basal laminal damage and subsequent fibrosis [29].

Few studies investigated mesothelial damage after pneumoperitoneum in humans [71, 72]. Tarhan et al. found mesothelial-cell apoptosis, cellular bulging up, intercellular clefts, and ultrastructural alterations in 20 biopsy specimens of the working area after laparoscopic cholecystectomy [72]. Therefore, this evidence highlights the role of different pneumoperitoneum parameters such as temperature, pressure, flow rate, and humidity in inducing mesothelial damage.

### 3.5. Adhesion Development

Peritoneal adhesions result in the abnormal repair of the peritoneum caused by surgical trauma, inflammation, and infection [62, 73, 74]. Consequently, the peritoneum promotes a healing process covering the damaged tissue with fibrin plug [75]. Normally, the fibrin deposit is entirely removed from the fibrinolytic system, and mesothelial regeneration is achieved in about 8 days [62,74]. Fibrinolytic activity can be compromised due to ischemic, inflammatory, or thermal insults causing fibroblast proliferation and adherence development [62,74].

During laparoscopy, the pneumoperitoneum can cause inflammatory phenomena that result in adhesions in relation to the type, temperature, and pressure of the insufflated gas and the length of pneumoperitoneum. Jacobi et al. observed a reduction in adhesions in mice undergoing laparoscopic colectomy using helium rather than CO_2_ [76].

If we consider the influence of intrabdominal pressure and the perfusion injury, we may speculate that increased intra-abdominal oxygen tension could be beneficial to the patient. In fact, in 2004, Binda et al. noted that hypothermia in an animal model prevented the formation of adhesions through a reduction in cellular oxygen needs. Subsequently, better results were obtained when humidifying the insufflated gas and adding 3% O_2_ [77]. Molinas et al. gained similar results in a prospective randomized trial. They supposed that the pneumoperitoneum can increase adhesion formation promoting hypoxemia due to the compression of capillary flow. The authors showed that intensifying the insufflation of CO_2_ or helium from 10 to 45 min increased the total adhesion score when adding 4% O_2_. No difference was observed between CO_2_ and helium [48]. In a randomized and controlled trial of 44 women undergoing deep endometriosis surgery, a decreased degree of adherence formation was found by inducing the pneumoperitoneum with a cooled and humidified gas composed of a mixture of 86% CO_2_, 10% N_2_O, and 4% O_2_ [78]. These findings are in accordance with the clinical report of reduced pain complaint, length of stay, and analgesic use in subjects receiving heated humidified CO2 compared to those treated with dry CO2 [79].

In 2007, Brokelman et al. reported that, in patients undergoing laparoscopic cholecystectomy using heated CO_2_, the level of plasminogen activator inhibitor type 1 (PAI-1) that hampered its activation was significantly reduced. Postoperative adhesions were thereby also reduced [80]. The same group observed a reduction in TGF*β*1, a PAI-1 promoter, when heating CO_2_ during pneumoperitoneum [81].

In addition to temperature, experimental data suggested that insufflation pressure can modify adhesions development. In 2011, Matsuzaki et al. compared the impact of intraperitoneal pressure of the pneumoperitoneum [82] in human and animal models. They measured the ratio of tissue plasminogen activator (tPA) and plasminogen activator inhibitor type 1 (PAI-1) in patients undergoing laparoscopic hysterectomy and subdivided them into two groups: standard-IPP (12 mmHg) and low-IPP (8 mmHg). After 2 hours of CO_2_ pneumoperitoneum, the tPA/PAI-1 ratio was significantly decreased in the standard-IPP group. Similar results were found in animal models with mice exposed to 2 mmHg (low IPP) and 8 mmHg (high IPP). Results showed that low IPP and the shorter length of laparoscopic surgery might reduce the impact of fibrinolytic activity and prevent the surgical adhesions development in the peritoneal cavity [82].

## 4. Conclusions

**Figure 3:**
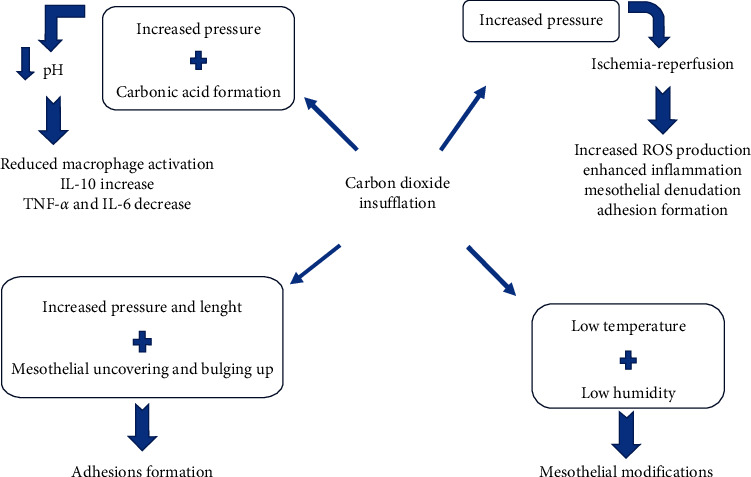
Summary of the mechanisms underlying peritoneal acidosis, oxidative stress, mesothelial alterations, and adhesions formation after CO_2_ insufflation.

In the era of minimally invasive surgery, knowledge of the basic physiological mechanisms that are modified by laparoscopy (Figure 3) and pneumoperitoneum is essential in making this surgical approach even less invasive. The actor responsible for these changes after laparoscopy is the lining that covers the abdominal cavity, the peritoneum, which maintains an extraordinary balance between all of its components and factors, such as chemokines, cytokines that play an important role in the activation and recruitment of leukocytes to inflammatory sites. Laparoscopy influences both peritoneal integrity and its biology, alters the immune system, and evokes peritoneal acidosis by CO_2_ insufflation and its influence on microcirculation. It is not clear how all these factors interact with each other and how they can be clinically translated, but knowledge of these mechanisms is the real challenge in truly minimally invasive surgery.
